# Self-Management Support to People with Type 2 Diabetes - A comparative study of Kaiser Permanente and the Danish Healthcare System

**DOI:** 10.1186/1472-6963-12-160

**Published:** 2012-06-14

**Authors:** Michaela Schiøtz, Martin Strandberg-Larsen, Anne Frølich, Allan Krasnik, Jim Bellows, Jette K Kristensen, Peter Vedsted, Peter Eskildsen, Henning Beck-Nielsen, John Hsu

**Affiliations:** 1 Section for Health Services Research, Department of Public Health, Faculty of Health Science, University of Copenhagen, Øster Farimagsgade 5, Building 10, DK-1014, Copenhagen k, Denmark; 2 Steno Health Promotion Center, Steno Diabetes Center, Niels Steensens Vej 8, DK-2820, Gentofte, Denmark; 3 Copenhagen Hospital Cooperation, Bispebjerg Bakke 23, Bispebjerg Hospital, 2400, Copenhagen, NV, Denmark; 4 Care Management Institute, Kaiser Permanente, One Kaiser Plaze 16th Floor, Oakland, CA 94612, USA; 5 Research Unit for General Practice, University of Aarhus, Bartholins Allé 2, DK-8000, Aarhus, Denmark; 6 Køge Hospital, Lykkebækvej 1, DK-4600, Køge, Denmark; 7 Department of Endocrinology M, Odense University Hospital, Kløvervænget 6, 4. Sal, DK-5000, Odense C, Denmark; 8 Mongan Institute for Health Policy, 50 Staniford Street, 9th Floor, Boston, MA, 02114, USA

**Keywords:** Self-management support, Type 2 diabetes mellitus, Health system, International comparison

## Abstract

**Background:**

Self-management support is considered to be an essential part of diabetes care. However, the implementation of self-management support within healthcare settings has appeared to be challenging and there is increased interest in “real world” best practice examples to guide policy efforts. In order to explore how different approaches to diabetes care and differences in management structure influence the provision of SMS we selected two healthcare systems that have shown to be comparable in terms of budget, benefits and entitlements. We compared the extent of SMS provided and the self-management behaviors of people living with diabetes in Kaiser Permanente (KP) and the Danish Healthcare System (DHS).

**Methods:**

Self-administered questionnaires were used to collect data from a random sample of 2,536 individuals with DM from KP and the DHS in 2006–2007 to compare the level of SMS provided in the two systems and identify disparities associated with educational attainment. The response rates were 75 % in the DHS and 56 % in KP. After adjusting for gender, age, educational level, and HbA1c level, multiple linear regression analyses determined the level of SMS provided and identified disparities associated with educational attainment.

**Results:**

Receipt of SMS varied substantially between the two systems. More people with diabetes in KP reported receiving all types of SMS and use of SMS tools compared to the DHS (*p* < .0001). Less than half of all respondents reported taking diabetes medication as prescribed and following national guidelines for exercise.

**Conclusions:**

Despite better SMS support in KP compared to the DHS, self-management remains an under-supported area of care for people receiving care for diabetes in the two health systems. Our study thereby suggests opportunity for improvements especially within the Danish healthcare system and systems adopting similar SMS support strategies.

## Background

The prevalence of type 2 diabetes mellitus (DM) is increasing rapidly and already comprises a major health burden globally [1,2]. Persons living with DM face substantial morbidity and mortality risks because of cardiovascular, renal, and neurologic complications. DM is a leading cause of end-stage renal failure, blindness, and limb amputation [3]. While there have been substantial improvements in the amount of knowledge concerning how best to treat patients with DM, including advancements in screening and pharmacologic therapies, patient self-care remains central to preventing complications. Not surprisingly, a number of organizations recommend providing self-management support (SMS) and view SMS provision as a necessary step for high-quality care [4,5].

It has been stressed that dissemination and implementation of SMS in the treatment of people with chronic conditions is not always easy or successful, [6] and several barriers affecting the implementation of SMS initiatives (e.g., lack of nurses, lack of integration of self-management support into standard care, and use of the traditional model of acute episodic care) have been identified in the literature [7,8]. However, research on how organizational structures influence the implementation of SMS is limited.

As many other European healthcare systems the Danish healthcare system (DHS) has targeted chronic condition care in its reform efforts. The DHS is recognized as having a reasonably good public system, but there is increasing recognition that the details of care organization and coordination are very important in order to provide high quality care. The DHS is a public financed system and belongs to the same family of healthcare systems as those of the other Scandinavian countries and the United Kingdom [9,10]. The US integrated healthcare system Kaiser Permanente (KP) has been recognized as a high-quality provider, especially for people with chronic conditions [11,12]. The Danish healthcare system has been shown to be somewhat comparable to KP in terms of budget, benefits and entitlements [13]. However, the delivery of diabetes care is managed and organized differently.

Comparative analysis is a powerful tool to highlight strengths and weaknesses in healthcare delivery systems [14,15]. Therefore, we compared the extent of SMS provided and the self-management behaviors of people living with diabetes in Kaiser Permanente (KP) and the Danish Healthcare System (DHS) in order to explore how different approaches to diabetes care and differences in management structure influence the provision of SMS. The objectives of our study were to investigate: 1) any differences between KP and the DHS in how the patient-specific processes of care that we describe as self-management support are provided; 2) if SMS was provided equally among socioeconomic groups; 3) and any differences between the systems in patient-reported self-management.

To set the scene for the comparative analysis we briefly present how diabetes care is provided in the two healthcare systems. KP screens members for diabetes, and structured medication management and education programs are implemented in KP as part of diabetes care [16]. All persons newly diagnosed with diabetes are offered diabetes education, which includes diet counseling and smoking cessation programs. Members with diabetes are typically followed up by their physicians every third month if their diabetes is in control. If there is a special need for control or if complications arise, patients can receive care management. The care manager acts as a facilitator, mentor, and guide through the diabetes care process and chooses appropriate medication according to a protocol that matches the patient’s blood tests, preferences, side effects, and history. Patients with serious conditions and/or several co-morbidities are supported by a case manager who ensures medical treatment and integration of care. KP’s integrated health information technology (HIT) system supports the provision of care by allowing for follow-up on clinical indicators over time, providing electronic reminders, and offering an up-to-date overview of available relevant services e.g. health education classes.

In the DHS, people with diabetes are treated in diabetes outpatient clinics, in family practice, or both, depending on the severity of the disease, the resources of the person with diabetes, and the professional interests of the family physician [17]. The care is provided according to national recommendations [18,19]. However, development of standards has typically not been followed with structured programs for cooperation, education, development of tools, and financial support. As a result, the care program offered in the DHS varies between counties, outpatient clinics, and family physicians. In some parts of the DHS, people with diabetes are offered diet counseling, diabetes-specific patient education, and, sometimes, psychosocial support and physical exercise programs. However, the package offered to people with diabetes depends on available resources, structure of care, and available services in the region. Disease management programs in the DHS are still in the initial phase of being implemented.

We hypothesized that the organization of care in KP improves the provision of SMS. Consequently, we expected to see a difference in the amount of professional support for self-management between KP and the DHS. Additionally, we hypothesized that the structured use of SMS in diabetes care would lead to better patient engagement and self-management and be equally applied among different socioeconomic groups.

## Methods

### Study population

Members with DM were randomly selected from the KP Care Management Institute (KP-CMI) established Clinical Outcomes Reporting and Evaluation (CORE) cohort. Cohort members were: 1) diagnosed with diabetes (both type 1 and type 2, as it was not possible to distinguish between the two types), 2) in contact with the health care system within the last six months, 3) current members of KP, and 4) between 18 and 75 years of age. Individuals on a “do not survey list” or without a valid address or phone number were excluded. The KP-CMI CORE cohort included people from all eight KP Regions. The Kaiser Foundation Research Institute Institutional Review Board (IRB) approved the study protocol. Because self-administered questionnaires were used to collect data, the IRB waived informed consent requirements, as is common practice. In 2005, an estimated 7.7 % of the KP adult population ages 20 and over had diabetes. Approximately 90 % of persons with diabetes in KP have type 2 diabetes.

Questionnaires were sent to 3,137 randomly-selected members of the CORE cohort in 2006, with a single follow-up mailing to non-respondents after two weeks that included another copy of the questionnaire. Non-respondents were telephoned and asked to complete the survey over the phone using Computer-Assisted Telephone Interviewing (CATI). Data was entered through the CATI software. The response rate was 56 %. In order to increase comparability to the Danish sample, only KP respondents between the ages of 40 and 75 years were included in the analysis; the final sample comprised 823 individuals.

**Figure 1:**
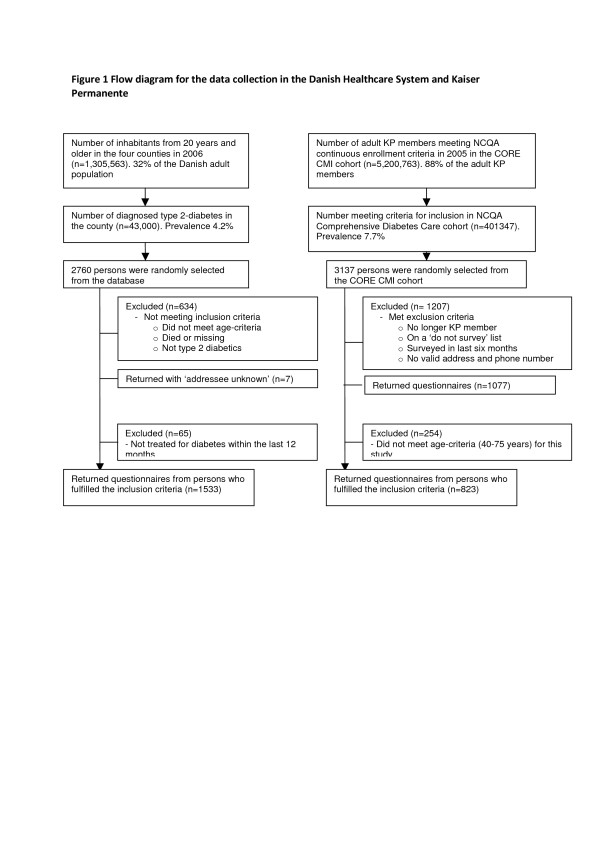
Flow diagram for the data collection in the Danish Healthcare System and Kaiser Permanente.

Danish people with DM were randomly selected from clinical databases that were available in four counties (Aarhus, Vejle, Roskilde and Funen). Inclusion criteria were: 1) confirmed DM diagnosis; 2) contact with the health care system within the last 12 months; 3) registration in a clinical diabetes database; and 4) between 40 and 75 years of age. It has been estimated that 4.2 % of the Danish population has type 2 diabetes [20]. People who participated in clinical trials were excluded because of the potential confounder of non-standard treatment. From the clinical databases containing 40,400 people with diabetes, 2,760 people from the four counties were randomly selected and sent a questionnaire in April, 2007. After two weeks, non-respondents received a mailed reminder. Four weeks after the initial questionnaires were mailed, non-respondents were again sent reminders, including another copy of the questionnaire. Overall, 1,598 individuals returned the questionnaire, reflecting a 75 % response rate (Figure 1). Under Danish law no ethical review process was required.

### Questionnaire development

The survey questionnaire was developed by a multidisciplinary work group from KP-CMI, based on a questionnaire originally developed in 2004 and augmented by validated items derived from a review of surveys (Commonwealth Fund International Health Policy Survey and the Commonwealth Fund International Health Policy Survey - Sicker Adults, the Patient Experience of Chronic Illness Care Survey, and the Consumer Assessment of Healthcare Providers Survey).

The questionnaire included items focusing on aspects of SMS, such as system support for collaborative goal setting (3 items), lifestyle management (3 items), medication management (4 items), and shared decision making (1 item); patient engagement, including the use of patient education programs (1 item), support groups (1 item), websites (1 item), and written information (1 item); and patient self-management behavior, including adherence to diabetes medication (1 item), exercise (1 item), and diet (1 item). Response options were largely yes/no or four-category scales (never/sometimes/usually/always); however, the medication adherence item had a seven-category response option (0 days to 7 days per week). Participants who responded that they “always“, “often” or “sometimes” experienced collaborative goal setting, life-style and medication management and shared decision making were categorized as receiving SMS according to international guidelines.

The Danish questionnaire was based on the KP-CMI survey questionnaire. To improve face and content validity, the questionnaire was translated and culturally adapted into Danish using a three-stage process [21]. Initially, two independent professional translators made forward-backward translations, and the questionnaire was pre-tested in groups of patients analogous to the survey population in terms of to age, gender, socio-economic status, and ethnicity. This assured that the respondents would not find the translated version of any item confusing, difficult to understand, ambiguous, or irritating. The collected data was double-keyed in using Epidata version 3.1.

### Statistical analysis

Compared to the survey sample in KP, members of the Danish survey sample were older, less educated, and included fewer women and fewer persons with a HbA1c level above 9 % (Table 1). Consequently, the analyses were adjusted for age, gender, HbA1c level, and educational level.

**Table 1:** Characteristics of Kaiser Permanente and Danish Healthcare System sample populations

	**DHS (n = 1533)**	**KP (n = 823)**
Mean age (95 % CI)	61.2 (60.9-61.6)	58.9 (58.3-59.5)
Female	38 %	52 %
Education		
Less than high school	32 %	8 %
Completed high school	26 %	44 %
Some college	27 %	18 %
College graduate	15 %	30 %
Mean BMI (95 % CI)	30.5 (30.2-30.8)	31.5 (30.9-31.9)
BMI above 30	48 %	50 %
Active smoker	25 %	7.7 %^1^
Mean HbA1c (95 % CI)	7.4 (7.3-7.5)	7.4 (7.3-7.5)
HbA1C > 8 %	23 %	24 %
HbA1C > 9 %	10 %	12 %

^1^Source: (Moffet et al. 2009).

We compared KP non-respondents to respondents in terms of age, gender, co-morbidities, hospitalization, and emergency department utilization. Respondents were older and more likely to have co-morbidities than were non-respondents (*p* < 0.001 for both).

In the two Danish counties (Vejle and Aarhus) where clinical data for non-respondents were available, we compared non-respondents to respondents in terms of age, gender, and hemoglobin A1c (HbA1c) level and found no significant differences for age and gender. However, non-respondents included 682 individuals whose HbA1c levels were lower than those of respondents.

Data from both systems were checked for missing data and outliers. KP and DHS respondents with missing items were compared to those without missing items in terms of gender, age, education, and HbA1c level. For the items with a considerable number of KP non-respondents, respondents and non-respondents did not differ according to age, gender and mean HbA1c level. For the Danish cohort, non-respondents were significantly older than respondents for items regarding collaborative goal setting, medication management and shared decision making (p ≤ 0.05). For all other items, non-respondents and respondents were similar.

All variables were tested for normal distribution and variance homogeneity and, where necessary, logarithmic transformations were used to achieve normal distributions. After adjusting for gender, age, educational level, and HbA1c, multiple linear regression analyses were used to compare the level of SMS received in the DHS and in KP. Multiple linear regression modeling was used to test disparities in received SMS according to educational attainment, age, and gender. SAS version 9.1 was used for the analyses, and *p* values < 0.05 were considered statistically significant.

## Results

SMS received varied substantially between the two systems (Table 2). KP respondents more frequently reported receiving all types of SMS for diabetes than did respondents from the DHS. The proportion of respondents reporting experience with collaborative goal setting, lifestyle and medication management, and shared decision making as part of their treatment was 29 % for KP and 13 % for the DHS (*p* < .0001). More people with diabetes in KP reported any use of SMS tools compared to DHS people with diabetes (75 % in KP and 47 % in the DHS) (Table 2).

**Table 2:** Self-management support in Kaiser Permanente and the Danish Healthcare System

**Aspects**	**Measure**	**Total n = 2256**	**DHS n = 1533**	**KP n = 823**	***p* value^a^**
Self-management support received	Always, often, or sometimes experienced collaborative goal setting, lifestyle, and medication management and shared decision making as part of their treatment	18 %	13 %	29 %	<0.0001
Collaborative goal setting	Discussed how to prevent illness with doctor	76 %	72 %	85 %	< 0.0001
	Plans and goals for treatment were explained	65 %	58 %	77 %	< 0.0001
	Received help to make an accurate plan to improve health	56 %	47 %	74 %	< 0.0001
Lifestyle and medication management	Discussed exercise, diet, and weight with doctor	85 %	85 %	85 %	0.413
	Adverse effects of prescribed medicine were explained within the last two years	37 %	26 %	56 %	< 0.0001
	Doctor revised medication, including medicine prescribed by other doctors, within the last two years	38 %	27 %	56 %	< 0.0001
	Spoke with doctor about the importance of taking prescribed medication	62 %	55 %	75 %	< 0.0001
Shared decision making	Doctor asked about preferred type of treatment or care if more than one option available	65 %	62 %	71 %	<0.001
	Discussed results of tests or examinations with health care provider	85 %	87 %	84 %	0.412
Use of SMS tools	Always, often, or sometimes used SMS tools	57 %	47 %	74 %	< 0.0001
	Used patient education or support groups^b^	18 %	11 %	30 %	< 0.0001
	Used websites with health information ^b^	28 %	28 %	30 %	0.364
	Used written health information ^b^	42 %	34 %	59 %	< 0.0001

^a^ Adjusted for age, gender, educational level, and HbA1c.

^b^ Within the last 12 months.

We found no statistically significant differences between SMS provided by healthcare professionals and educational attainment within KP. However, significantly more people in KP with a high educational level reported using websites compared to people with a low educational level.

For the Danish respondents, we found no differences among educational attainment groups for most of the surveyed items. However, 96 % of people with the most education reported that they had discussed with their doctor things they could do to prevent a deterioration of their disease, compared with 80 % of people with the lowest education level (p = 0.006). Similarly, 91 % of people with the most education reported that they always or often had discussed the results of tests or examinations with their provider, compared 85 % of people with the least education (p = 0.018). Conversely, 60 % of people with the least education had talked with their doctor about the importance of taking prescribed medications, compared to 51 % of people with the highest educational (p = 0.022). With regard to use of self-management tools, among people with the most education, 45 % used websites with health information and 43 % used written material about self-management of their disease, compared to the group with the least education, where the comparable percentages were 16 % and 28 %, respectively. Reported adherence to diabetes medication was high in both systems (77 % in KP and 90 % in the DHS), while only about half of the survey population reported that they followed the national recommendations regarding exercise (Table 3). Less than half of the respondents reported that they adhered to both prescribed medication and national recommendations for exercise (45 % in KP and 46 % in the DHS).

**Table 3:** Self-management behavior in Kaiser Permanente and Danish Healthcare System

**Behavior**	**Total n = 2256**	**DHS n = 1533**	**KPn = 823**	***p* value^a^**
Followed a regular exercise schedule within the last 12 months.	58 %	56 %	60 %	0.378
Took prescribed medication every day previous seven days	85 %	90 %	77 %	< 0.0001
Follow national exercise guidelines and took prescribed medication seven days a week	47 %	46 %	45 %	0.457

^a^ Adjusted for age, gender, educational level, and HbA1.

The differences between systems were present in both high and low socioeconomic strata, meaning that even though more people with a high education in the DHS received support for some aspects of self management, they still received less SMS than did the similar segment of the KP respondents.

## Discussion

We compared representative samples of the KP and DHS diabetes populations and found significant differences in received SMS. People with diabetes in KP reported that they received substantially more SMS, compared to the Danish population. However, received SMS was not optimal in either system. One third of the KP respondents received the level of SMS recommended in international guidelines, compared to only one in 10 DHS respondents.

In addition, more KP patients used SMS tools than did those in the DHS; three out of four respondents had used SMS tools within the last 12 months (e.g., patient education, support groups or websites, or written information about health and diseases). There were more men in the Danish sample population (62 %) than in the sample population from KP (48 %). Males are known not to use self-management tools to the same degree as women [22]. Thus, the level of use of SMS tools in the DHS population could have been higher if the sample had included more women. In KP, received SMS did not vary between different educational groups. This was not the case in the DHS, where a greater proportion of people with the highest amount of education received SMS and discussed their medical test results with their doctor, compared to the group with the least amount of education. Conversely, more people in the DHS with the least amount of education discussed the importance of medication adherence with their doctor. An explanation for the variation in SMS provided to different educational groups in the DHS and lack of difference between SMS provided to different educational groups in KP could be the significantly lower educational attainment of the DHS population compared to the KP population. Another potential explanation for the variation in SMS provided to different educational groups in the DHS may be that there is less focus on tailoring care to specific groups in a welfare system like Denmark’s, which has relatively low social disparities compared to the US. However, Kaiser members also tend to come from middle to mid-lower socioeconomic group because wealthier families mostly opt for more flexible and more expensive healthcare options. Thus, the KP population also has relatively low social disparities compare to the general US population. Prior studies found that lower education is associated with poorer diabetes-related health behaviors [23-25]. Karter et al. (2007) concluded that, during the course of life, the cumulative effect of reduced practice of multiple self-care behaviors among less educated patients may play an important part in shaping the social health gradient.

With respect to self-management behavior, less than half of the respondents in both systems reported that they exercised as recommended in national guidelines and adhered to prescribed diabetes medication. The cross-sectional study design prevents us from concluding whether self-management behavior in the two populations are linked to HbA1c levels and self-management support, although prior research shows that education and physical activity improve blood glucose regulation [26,27]. Furthermore, medication (and, by obvious implication, medication adherence) has a significant effect on regulation of blood glucose levels. The populations’ mean HbA1c level may also be influenced by treatment intensity, disease severity, and measurement/monitoring practices. However, in order to investigate these relationships, longitudinal experimental studies or quasi-experimental follow-up studies are needed.

When conducting comparative analysis it is important to be aware that the specific configurations of any healthcare system depend on the historical and cultural context of health and healthcare that varies across and within countries when conducting comparative research [28-30]. When engaging in a cross-sectional, comparative study there are therefore potential lessons to be learned but also methodological challenges and results should therefore be interpreted with care. Strengths of our study include two relatively large samples of individuals with diabetes randomly selected from two different well-described health care systems. Furthermore, the data was collected with a questionnaire developed specifically to capture dimensions of SMS and behavior in a diabetes population.

A limitation to our study is that the inclusion criteria for the two study populations differed, as both people with both type 1 and type 2 diabetes were included in the KP survey and only those with type 2 diabetes were included in the Danish survey. However, 90 % of the diabetes population in KP has type 2 diabetes, and the recommendations regarding SMS are comparable for type 1 and type 2 diabetes. Therefore, we can assume that the responses regarding SMS do not differ significantly between people with type 1 and type 2 diabetes and, consequently, do not explain the differences in SMS between the two systems.

A more relevant difference between the two systems is that KP screens members at risk for diabetes, meaning that people with undiagnosed diabetes will be identified earlier and that diabetes care, including SMS, can be initiated earlier than is the case in the DHS. To some extent, this may explain the different levels in the prevalence of diagnosed type 2 diabetes: 7.7 % in KP compared to 4.2 % in the DHS. The level of SMS support may vary according to duration of disease, as people newly diagnosed with diabetes may receive more education and support than people who have lived with diabetes for many years. It was not possible to obtain information about duration of disease from KP, but we can assume that the survey sample in KP has, on average, been diagnosed and treated for diabetes longer than the Danish survey sample. We surmise that, if it had been possible to adjust our analysis for disease duration, we would have found a greater difference between the systems in the level of SMS provided.

Another limitation of the study is the questionnaire used. The questionnaire was developed for use in a sample population from a US integrated healthcare plan and was not constructed to be used in a comparative analysis of health care systems. Thus, some of the questions may have described more accurate the way SMS is provided in KP and less accurate how SMS is provided in the DHS. Most likely this means that the questionnaire has captured SMS provided in KP to a greater extend than SMS provided in the DHS. It can also be questioned whether the differences in reported SMS between the two health care systems can be explained by differences in interpretation of SMS. However, we asked about specific activities related to SMS to make it more likely that the understanding of the questions would be consistent in the two cultures. For example, the participants were asked how often their regular doctor helped them with a specific plan for what they could do to improve their own health. For both systems, the questionnaire was subjected to extensive cognitive testing to ensure that the questions were easy to understand and understood as intended after being translated from English to Danish using a two-stage process. The surveys were designed for an eighth-grade reading level.

Another potential limitation to our study is self-selection bias. The non-respondent rate was substantially higher in the KP survey sample. Non-respondents had more co-morbid conditions than did respondents, and it is possible that individuals with multiple co-morbidities receive more SMS support. The level of SMS reported as received might have been lower in KP if non-respondents had also participated in the survey.

Despite the minor differences between the two populations and the limitations of the study design, we believe that the results reflect real variations in the level of SMS provided in the two health systems. Furthermore, we believe that the differences in extent and distribution of SMS between the systems can be attributed by differences in the organization of care delivery. This includes the systematic approach to diabetes care including SMS in KP which comprises use of clinical guidelines, stratification of patients according to need for care and support, use of the integrated HIT system allowing for systematical follow-up on patients, panel management and an overview of available SMS services within the healthcare system. Further research is needed to examine how such approaches will influence the delivery of chronic care in public financed healthcare systems.

## Conclusion

While patient self-management is recommended as part of diabetes care, relatively few people with diabetes receive support from health care professionals for these efforts. We found substantial differences in SMS reported as received across the two health care systems. For most aspects of SMS, KP performed better that the DHS. This pattern was also reflected in the use of SMS tools, but not in relation to other self-management behaviors. The adherence to diabetes medication was relatively high but adherence to national exercise recommendations was relatively low in both systems.

Patient self-management represents an important but under-supported area of care for those with chronic conditions. Efforts to improve SMS could help address quality concerns in healthcare systems like the DHS. This may includes structured approaches to the delivery of care including use of clinical guidelines, stratification of patients depending on needs for care and support, systematic follow-up of patients and development tools to provide SMS.

Additional research is needed to assess the organizational and patient factors associated with greater SMS use, effective approaches for delivering the support, and the link between SMS and health outcomes.

## Competing interests

Martin Strandberg-Larsen is an employee at Novo Nordisk A/S. The remaining authors declare that they have no competing interests.

## Authors’ contributions

MS designed the concept and conducts of the study, collected analysed and interpreted data, and drafted the manuscript. MSL collected, analysed, and interpreted data and helped to draft the manuscript. AF, AK and JB assisted in designing the study, data collection and interpretation of data and helped to draft the manuscript. JKK, PV, PE, and HBN provided data to the study, and assisted in data interpretation and drafting the manuscript. JH assisted in designing the study, analysed and interpreted data and provided a critical revision of the manuscript for important intellectual matters. All authors read and approved the final manuscript.

## Pre-publication history

The pre-publication history for this paper can be accessed here:

http://www.biomedcentral.com/1472-6963/12/160/prepub
